# Device aided therapies in Parkinson disease: an expert view on apomorphine

**DOI:** 10.1007/s10072-026-09228-5

**Published:** 2026-07-15

**Authors:** Fabrizio Stocchi, Roberto Ceravolo, Carlo Colosimo, Maria Francesca De Pandis, Nicola Modugno, Manuela Pilleri, Alessandro Tessitore, Michele Tinazzi, Franco Valzania, Carmine Vitale, Roberta Zangaglia, Mario Zappia, Maurizio Zibetti, Angelo Antonini

**Affiliations:** 1 Department of Neurology, University San Raffaele, Rome, Italy; 2 Institute for Research and Medical Care, IRCCS San Raffaele, Rome, Italy; 3 Department of Clinical and Experimental Medicine, Neurology Unit, University of Pisa, Pisa, Italy; 4 Department of Neurology, Santa Maria University Hospital, Terni, Italy; 5 IRCCS San Raffaele Roma, Cassino, Italy; 6 Department of Human Science and Promotion of Quality of Life, San Raffaele University, Rome, Italy; 7 I.R.C.C.S. Neuromed, Pozzilli, Isernia Italy; 8 UO Neurologia Casa Di Cura Villa Margherita, Arcugnano, Vicenza, Italy; 9 Centro Parkinson E Parkinsonismi, ASST Gaetano Pini CTO, Milan, Italy; 10 Department of Advanced Medical and Surgical Sciences, University of Campania “L. Vanvitelli”, Naples, Italy; 11 Department of Neurosciences, Biomedicine and Movement Sciences, University of Verona, Verona, Italy; 12 Neuromotor and Rehabilitation Department, Neurology Unit, Azienda AUSL-IRCCS Di Reggio Emilia, Reggio Emilia, Italy; 13 Department of Medical, Motor and Wellness Sciences, University of Naples “Parthenope”, Naples, Italy; 14 Parkinson’s and Movement Disorders Center, ICS Maugeri Hermitage, Naples, Italy; 15 Parkinson’s Disease and Movement Disorders Unit, National Institute of Neurology, IRCCS “C. Mondino Foundation”, Pavia, Italy; 16 Department of Medical, Surgical Sciences and Advanced Technologies “GF Ingrassia”, University of Catania, Catania, Italy; 17 Department of Neuroscience “Rita Levi Montalcini”, University of Turin, Via Cherasco 15, 10126 Turin, Italy; 18 SC Neurologia 2U, A.O.U. Città Della Salute E Della Scienza Di Torino, Corso Bramante 88, 10126 Turin, Italy; 19 Neurodegenerative Disease Unit, Department of Neuroscience, Padua Neuroscience Center (PNC), University of Padua, Padua, Italy; 20 IRCCS, San Camillo Hospital, Venice, Italy

**Keywords:** Advanced Parkinson’s disease, Apomorphine, Continuous apomorphine infusion, Device-aided therapies, Levodopa, Parkinson’s disease

## Abstract

**Introduction:**

Parkinson’s disease (PD) affects approximately 1% of individuals over the age of 60, with its prevalence increasing in older populations. Clinical manifestations can be effectively controlled during the first years of disease but progressively medications effect shortens with appearance of motor complications and dyskinesia.

**Areas covered:**

Management of PD primarily aims to restore dopaminergic activity through pharmacological interventions such as levodopa, which effectively alleviates motor symptoms and extends life expectancy. Early initiation of treatment improves bradykinesia, rigidity, and tremor; however, as the disease advances, the therapeutic response to levodopa shortens, leading to complications such as motor fluctuations and dyskinesia. In advanced Parkinson’s disease (aPD), the emergence of the “wearing-off” phenomenon and unpredictable “on/off” fluctuations—where symptoms reappear despite medications—poses significant challenges for disease management.

**Expert opinion:**

Device-aided therapies, including deep brain stimulation and continuous infusion of apomorphine or levodopa, provide effective management options for advanced Parkinson’s disease (aPD) when oral medications fail to adequately control symptoms. Continuous subcutaneous apomorphine infusion (CSAI) has demonstrated both efficacy and long-term tolerability; however, its clinical adoption remains limited due to challenges in patient selection and referral, treatment initiation and handling of the device. This review examines the available infusion therapies, with a particular focus on CSAI, and aims to provide clinicians with evidence-based guidance for optimizing treatment selection in aPD.

## Introduction

Parkinson’s disease (PD) affects 1% of the population aged over 60 years and can present in up to 3% in older age groups [1, 2]. The neuropathological hallmarks reflect the loss of dopaminergic neurons in the substantia nigra pars compacta area of the basal ganglia, resulting in a deficiency of dopamine and in a downregulation of the dopaminergic neural circuits. A characteristic neuropathological signature is the accumulation of alpha-synuclein aggregates in the dopaminergic neurons. Thus, PD as a clinical syndrome is defined by a combination of motor problems-namely, bradykinesia, rigidity, resting tremor, flexed posture, "freezing," and loss of postural reflexes. Moreover, non-motor symptoms such as depression, dementia, apathy and dysautonomia are a common feature of PD patients [1].

In the absence of treatment, PD progresses to an akinetic state, where patients are unable to care for themselves, and death is primarily attributed to complications arising from the disease [1]. Management strategies for PD have primarily focused on restoring dopaminergic activity through the use of levodopa, enzyme inhibitors, and dopamine agonists, which have proven effective in alleviating most clinical symptoms and delaying disease progression. With appropriate pharmacological treatment, functional mobility can be preserved for many years, and life expectancy is significantly extended in adequately treated patients [1].

PD treatment strategies vary depending on patient characteristics, symptom presentation, and individual responses to specific medications. Effective management requires a personalized approach, with periodic reassessments as the disease evolves [2]. Early in the disease course, dopamine replacement therapies lead to dramatic improvements in bradykinesia, rigidity, and tremor [3]. Symptoms are primarily managed with oral medications, such as levodopa-carbidopa, often combined with dopamine agonists, Catechol-O-Methyl-Transferase (COMT) inhibitors, and Monoamine Oxidase B (MAO-B) inhibitors [4–6].

However, as the disease progresses, levodopa treatment becomes less effective, and complications such as motor fluctuations and dyskinesia arise [7]. The progression of PD results in the worsening of both motor and functional symptoms, which, coupled with motor and non-motor complications, significantly impacts the patient’s quality of life. Conventional medications lose their effectiveness, failing to provide adequate symptom control; this later stage is referred to as advanced Parkinson’s disease (aPD) [8].

A major challenge in managing aPD is the development of fluctuations specifically the “wearing off” phenomenon [8], where levodopa treatment only provides symptom relief for a brief period, typically two to three hours. Delayed ON or NO-ON episodes, where levodopa either takes longer than expected to exert its effects or fails to work altogether, further complicate management [9]. As a result, akinesia and rigidity rapidly return toward the end of the dosing interval and may not improve after subsequent levodopa administration. This leads to fluctuations between "off" periods, during which medication fails to provide therapeutic benefit, and "on" periods, where patients experience dyskinesia, a phenomenon known as the "on/off" cycle.

Managing symptoms of aPD presents significant challenges, particularly in optimizing oral therapies (especially polypharmacotherapy) and utilizing device-aided therapies (DAT), such as deep brain stimulation (DBS), continuous subcutaneous apomorphine infusion (CSAI), levodopa-carbidopa intestinal gel (LCIG) infusion, or subcutaneous levodopa infusion [9]. Moreover, MRI-guided focused ultrasound (MRgFUS) and extended-release carbidopa/levodopa formulations are proving to be of use in PD treatment. They address motor fluctuations and tremors, allowing for longer symptom control and improved daily functioning [10–12]. Notably, inclusion in the aPD category does not automatically warrant the use of DAT; rather, patients selected for DAT are a subset of the broader PD population. Furthermore, the optimal timing for initiating these treatments and selecting the most appropriate therapy remains uncertain [13, 14]. Moreover, it is recognized that the female population is less well represented in clinical trials on infusion therapies for the management of aPD [15], thereby making the management of this category of patients more complex.

Given the lack of a consensus on the management of aPD and what could be the more appropriate DAT, a better understanding of the differences among available DAT is crucial. A panel of movement disorders experts met with the aim to highlight patient characteristics that drive eligibility for DAT and may help establish a patient profile that could most benefit from them. Thus, this expert opinion paper is a narrative review that revises evidence on the currently available infusion therapies as advanced therapies for the management of aPD, with a focus on continuous apomorphine infusion. Furthermore, it aims to clarify the criteria that allow the best patient management, giving a practical guidance for initiation of apomorphine infusion therapy.

## Patient’s selection for DAT

As disease progresses and reached aPD stage, the beneficial response to symptoms shortens and patients experience the on/off periods [16]. Approaches to overcome the fluctuations include adding a medication (DA agonist, MAOBI, COMTI, A2A antagonist), shortening dosages intervals of L-dopa, or introducing a long-acting L-dopa preparation where available [17, 18]. One of the challenges in choosing the correct timing to switch to DAT is the lack of a consensus on how to define the stage of aPD [19]. Moreover, the H&Y scale is usually used in clinical settings, but it does not take into account motor complications and non-motor symptoms which are considered key elements to determine disease progression [20]. Other scales have been developed, such as the Parkinson’s disease composite scale (PDCS), which consider severity of motor and non-motor symptoms, and complications of long-term symptoms. However, this scale is not widely used [18]. On the other hand, associating Movement Disorder Society – Unified Parkinson's Disease Rating Scale (MDS-UPDRS) motor scores to disease severity requires the intervention of movement disorders specialists with specific expertise and the reality of clinical practice does not always allow this comprehensive patient evaluation, due to busy clinical workflows [21].

**Fig. 1:**
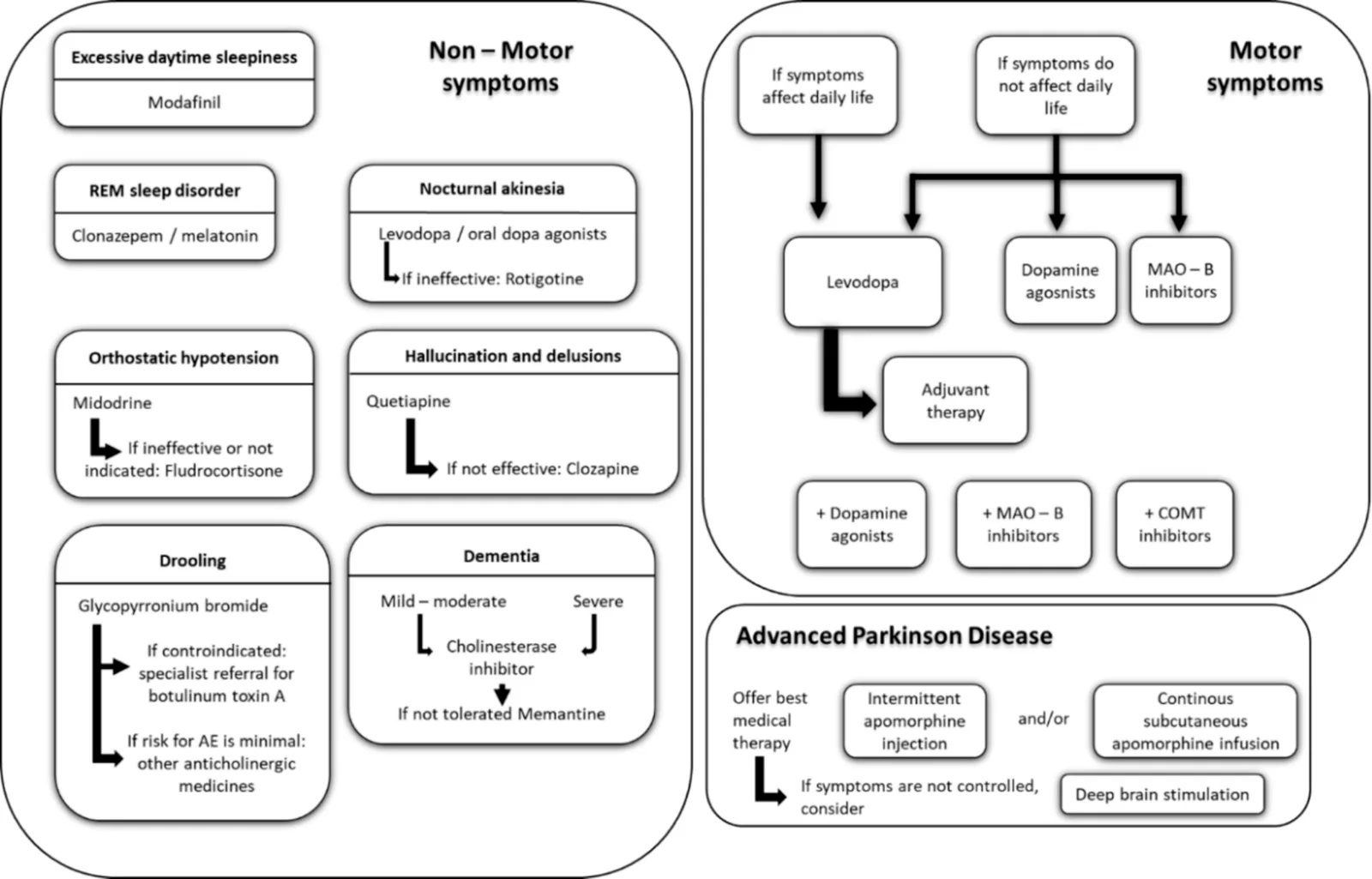
The most recent update to the National Institute for Health and Care Excellence (NICE) guideline for the diagnosis and management of Parkinson’s disease in adults provides an update on most aspects of managing Parkinson’s disease

The most recent update to the National Institute for Health and Care Excellence (NICE) guideline for the diagnosis and management of PD in adults provides an update on most aspects of managing PD (Fig. 1). However, it is still unclear how best to identify aPD patients that may benefit from DAT and what is the best DAT to use in the aPD patient.

## Infusion therapies for the management of APD

It is well established that oral levodopa bioavailability is affected by its administration route and pharmacokinetics [22, 23]. As mentioned above, the current management of aPD is mainly based on improving dopaminergic tone [19]. DAT offer reliable alternative solutions that may overcome the issues by delivering a continuous dopaminergic stimulation in the substantia nigra pars compacta neurons (Table 1). In fact, the rationale for infusion therapies is to provide a continuous drug delivery to achieve continuous dopaminergic stimulation. Different studies demonstrated how constant plasma levels of L-dopa or of apomorphine can reduce the impact of adverse events and overall improve patient management through better compliance and reduction of dopaminergic hypersensitivity [19, 24–26].

**Table 1:** Drug summary box

Drug name	Apomorphine hydrochloride
Phase	Phase 4
Indication	Treatment of motor fluctuations ("on/off" phenomena) in patients with Parkinson's disease who are not sufficiently controlled by oral anti-Parkinson medications
Pharmacology	Dopamine receptor agonist (all subtypes of D1 and D2 receptors) and affinity for serotonergic receptors 5HT2A, 5HT2B, 5HT2C and adrenergic receptors α2A, α2B, α2C
Route of administration	Subcutaneous administration
Chemical Structure	Tetracylcine aporphine ring
Pivotal trials	APO-NEO (Apomorphine Neon) Study; STUDY 021; APO-SYN (Apomorphine Subcutaneous Infusion Study); ADVANCE-PD

### Duodenal L-dopa infusion

Oral L-dopa has been the gold standard in the management of PD. However, issues related to its pharmacokinetic profiles soon emerged and other administration route were explored.

Duodenal L-dopa infusion aims at bypassing the gastric emptying, which is responsible for the drug’s oscillation during oral therapy and allows to achieve continuous L-dopa delivery with an optimized dose that can be kept stable. On the other hand, this approach was clinically less tolerated due to the irritative nature of L-dopa to the vasculature. Thus, L-dopa/carbidopa gel formulation was developed [26]. LCIG bypasses the stomach through an intrajejunal percutaneous tube connected to an externally carried pump, thus providing direct access of the drug to the small intestine, where the ammino acid transporter is concentrated, leading to more stable plasmatic concentrations of the drug [26–28]. LCIG requires placement of a percutaneous endoscopic gastrostomy-jejunal tube (PEG-J). Titration of LCIG is performed for a few days after insertion and dosage is adjusted based on the patient’s clinical response, while oral medications are slowly discontinued [29].

Many real-world evidence studies showed the efficacy of LCIG on improving motor profile [30, 31]. Moreover, randomized clinical trials showed the superiority of LCIG over standard of care (SOC) [32]. Long-term data confirmed the benefits overtime, reducing the OFF times and improving quality of life [33, 34].

### Continuous subcutaneous apomorphine infusion (CSAI)

Over the years, apomorphine has been widely used in medicine, showing a range of effects on the central nervous system and already in 1884, Weil begun to use it in PD patients [35, 36].

Apomorphine is an aporphine alkaloid, which is obtained from morphine acidification. The chemical structure consists of a tetracycline aporphine ring, which gives the molecule a lipophilic profile and allows the recognition of dopaminergic receptors. It acts as a powerful dopamine receptor agonist, with affinity for all the subtypes of D1 and D2 receptors (D1, D2S, D2L, D3, D4, D5) [37]. Moreover, apomorphine shares some affinity for the serotonergic receptors 5HT2A, 5HT2B and 5HT2C and adrenergic receptors α2A, α2B and α2C [37]. On the other hand, apomorphine does not bind to opioid receptors [38]. The very low oral bioavailability of apomorphine led to the development of alternative parental routes of administration and the subcutaneous route is the one used in the management of aPD either as acute bolus or continuous subcutaneous infusion. The clinical efficacy has been confirmed in many studies, where apomorphine reversed sever and sudden OFF states in aPD, despite optimal oral therapy [39–41], and even when CSAI was used as monotherapy [42]. This route of administration has some limits linked to various factors that may hamper the absorption (site of injection, presence of nodules, quantity of body fat, and state of the skin) [1, 43]. Continuous delivery of apomorphine can be ensured through delivery of the drug in a pre-filled vial attached to a pump and secure to a subcutaneous needle [44]. Apomorphine can be titrated in an inpatient or outpatient setting by either starting with the lowest infusion rate and slowly increasing it by 0.5–1 mg/h on a daily to weekly basis. Based on the clinical response, oral L-dopa and other medications are reduced up to 50% [44].

The continuous infusion of apomorphine allows a rapid onset of clinical response since the first or second day of infusion, as demonstrated in many clinical studies in PD patients and in a large multicenter controlled trial [45]. In fact, when used continuously, apomorphine is able to markedly reduce up to 2 h the daily OFF time, increase by up to 2 h daily ON time without troublesome dyskinesia and allows a reduction in oral medications [31, 45].

Moreover, recent studies in selected patients who experience severe night symptoms showed a benefit in extending the treatment up to 24 h, provided that there is a treatment-free interval of at least 24 h [46].

### Continuous subcutaneous infusion of Foslevodopa/Foscarbidopa

LC‑CSCI therapy has a similar delivery system to CSAI and consists of a soluble formulation of levodopa/carbidopa delivered subcutaneously by a portable pump. Two molecules have completed clinical development: ND0612 [47, 48] and ABBV-951 [49–51].

ND0612 is a drug-device combination consisting of a sterile solution of levodopa/carbidopa continuously delivered via a dedicated subcutaneous pump [47]. ND0612 has an international, multicenter, 102-month, open-label study ongoing (BeyoND study, NCT02726386), that will be concluded in 2027. Preliminary data have already been published and, while the clinical efficacy was reached, 85.5% of patients showed at least one AE, even if of mild-to-moderate entity and mostly related to the site of injection. In fact, AE led to discontinuation of treatment in 17.3% of patients [52].

ABBV-951 is made up of two prodrugs foslevodopa/foscarbidopa, which are converted to levodopa/carbidopa after administration by alkaline phosphatases, reaching an optimal plasma concentration in rapid time. This formulation can be delivered subcutaneously for up to 24 h/day.

The continuous subcutaneous infusion emerges as an innovative treatment, which may allow a sustained symptom control, overcoming the limitations associated with the other alternatives [51].

Foslevodopa and foscarbidopa are prodrugs of levodopa and carbidopa, respectively. Their chemical structure showed high stability in a wide pH range and are rapidly converted to their active forms predominantly in the liver [53, 54]. Pharmacokinetic studies demonstrated how a continuous subcutaneous infusion of foslevodopa-foscarbidopa (ratio 4:1) allowed to reach plasma concentrations of 1.5–4.5 μg/mL, respectively. These concentrations are within therapeutic range for most patients with aPD [50].

Clinical efficacy was evaluated in a phase III RCT, where foslevodopa-foscarbidopa was administered as a continuous subcutaneous infusion over 12 weeks and it was compared against oral levodopa-carbidopa [51]. The foslevodopo-foscarbidopa group reached the primary endpoint, with a significant improvement in “on time” without dyskinesia as compared to the control group (2.72 ± 0.52 h as compared to a 0.97 ± 0.50 h, respectively. 95% CI = 0.46–3.05; P = 0.0083). In parallel, there was a significant reduction in the “off time” in the foslevodopo-foscarbidopa group as compared to the control group (2.75 ± 0.50 h vs 0.96 ± 0.49 h, respectively. 95% CI = 3.03 to 0.54; P = 0.0054). [51].

Another open-label trial confirmed a significantly superior efficacy of the continuous subcutaneous infusion of foslevodopo-foscarbidopa as compared to oral medication in patients with PD [55].

### Levodopa/entacapone/carbidopa intestinal gel (LECIG)

LECIG intestinal gel has been developed combining L-Dopa carbidopa and entacapone (LECIG). The inclusion of entacapone in the LECIG formulation extends the clinical benefits of LCIG allowing the administration of a reduced levodopa dose to achieve the same levodopa exposure, while also reducing levels of potentially harmful levodopa metabolites, such as 3-O-methyldopa and possibly homocysteine. Moreover LECIG can be delivered through a Crono LECIG pump, which is substantially smaller of the CAFF Legacy 1440 used to infuse LCIG solution. Clinical studies suggest that LECIG is as effective as LCIG and well tolerated by PD patients, with a safety profile similar to that of LCIG infusion plus oral entacapone and patients are reporting that they prefer the reduced pump size and weight; LECIG therefore offers another useful DAT option to consider for suitable people with advanced PD who may need intrajejunal levodopa therapy [56].

To date, clinical experience is limited and reported improvement in motor symptoms and quality of life [57]. Pharmacokinetic studies showed comparable levels of L-dopa after exposure to LCIG and LECIG, with a 20% reduction in infusion dose vs LCIG [58]. A large multicenter international, prospective, non-interventional, observational study (ELEGANCE) evaluating the use of LECIG for up to 12 months demonstrated sustained control of motor fluctuations, similar safety profile to LCIG and a positive impact on QoL [59].

## Safety of DAT

Overall, DAT are well tolerated and show safety profiles that are similar to SOC, with some peculiar differences when considering different DAT.

For instance, LCIG has shown to be a valid alternative to SOC in terms of efficacy. However, the safety profile may show a higher rate of adverse events. The most frequent complications include weight loss and abdominal pain, as well as neuropathy. Specifically, peripheral neuropathy represents a well-recognized complication of LCIG therapy, occurring in approximately 10–15% of treated patients, with elevated plasma homocysteine and altered vitamin B levels identified as key pathophysiological mediators; vitamin B supplementation appears to reduce the coupling between levodopa dose and homocysteine and may help prevent LCIG-related polyneuropathy [60]. On the other hand, serious AE can show in up to 53% of cases, with a small percentage that may experience severe AE. In long-term studies with average follow-up of 4 years, reported tube replacements in 30% of cases [34]. Overall, interruption of therapy is most frequently related to AE and ranges from 3 to 24% [32, 35].

On the other hand, CSAI is usually well tolerated, and AE are mainly linked to the administration route and include skin reactions and nodule formation, with higher incidence in patients receiving CSAI rather than in those receiving intermittent injections (70% vs 11%, respectively) [61]. Other AE are nausea and somnolence (13% vs 4%, respectively), which can be easily controlled with the administration of other drugs (antiemetics such as domperidone or trimethobenzamide) during titration period and until symptoms cessation [62]. Central dopaminergic AE are mostly sedative effects, whereas confusion, hallucinations and psychosis are less commonly observed, probably due to different receptors affinity [63].

Hematologic AE are a rare complication; however, autoimmune hemolytic anemia may develop, and the risk should be carefully assessed in patients undertaking CSAI treatment through monitoring of hemolytic parameters and the detection of anti-red blood cells antibodies with the Coombs test [64]. Moreover, cardiovascular risk of QT/QTc interval prolongation should be evaluated, especially when apomorphine is given in combination with domperidone in patients with preexisting cardiac disease [65].

After reaching optimal dosage of subcutaneous apomorphine (usually between 4 and 7 mg/hour), patients may continue on stable doses for many years. Moreover, being a subcutaneous administration, it does not require any surgical intervention and is easily reversible [44, 66].

In preclinical studies, in patients treated with continuous subcutaneous infusion of Foslevodopa/Foscarbidopa (CSCI), the occurrence of adverse skin reactions was frequent [50, 54], which was confirmed in the clinical studies [51, 55], even if they were classified as mild to moderate, manageable with appropriate care and did not lead to treatment discontinuation. Notable, in both studies the catheter was left in place for 1 to 3 days, which could explain the high rate of adverse reactions.

Another common reported side effect was nausea and vomiting, reported at a higher rate than in the control group (45% vs 38%, respectively) [51]. On the other hand, serious adverse events were comparable between the two groups.

Approaches aimed at mitigating the common side effects should be carefully evaluated to maximize the clinical benefit of this new alternative and avoid treatment discontinuation. In fact, Soileau et al. noted a treatment discontinuation linked to adverse event sin 22% of patients as compared to 1% of patients in the control group, highlighting the severity of the impact of adverse events on patient compliance and quality of life [51]. Further studies are currently undergoing for both alternatives, especially for the evaluation of long-term efficacy and safety.

Notable, long-term adherence to subcutaneous infusion therapies may represent a clinically relevant challenge: approximately 35% of patients discontinue CSAI within two years of treatment initiation, and the minimally invasive and reversible nature of apomorphine infusion, while advantageous, may paradoxically contribute to its high dropout rate, as difficulties are more readily acted upon compared to more invasive DAT [67, 68]. Similarly, the discontinuation rate due to adverse events with CSCI foslevodopa/foscarbidopa has been reported at 22%, notably higher than that observed with oral levodopa/carbidopa, raising concerns about long-term tolerability [69]. Beyond adverse events, patient autonomy represents a key driver of non-adherence, as the continuous connection to an infusion pump may be particularly burdensome for younger, more active patients, who may subsequently transition to DBS. In this context, it is crucial the role of care-givers to reach a successful treatment management [67–69].

Notably, not many head-to-head comparison data or systematic reviews are available on the topic, especially if considering also non-infusion therapies. A Bayesian network meta-analysis by Antonini et al. demonstrated that, among DAT for aPD, LCIG and DBS were associated with superior improvement in off-time and PD-related quality of life compared with CSAI and best medical therapy at 6 months after treatment initiation [12].

## Expert opinion: what therapy to choose and how to initiate it

DAT provide an opportunity to manage aPD when oral medications fail to adequately control motor fluctuations and dyskinesia or when the treatment regimen becomes excessively complex for the patient.

While DAT options share similar indications, most patients may benefit from more than one modality, necessitating the development of clinical parameters to guide therapy selection. Despite existing regulations, clinical practice often relies on physician experience and habitual decision-making rather than robust scientific evidence. Additional factors, such as the impact of DAT on non-motor symptoms and overall quality of life—particularly regarding device-related comfort—should also be considered [70].

Recent consensus findings suggest that clinical and demographic factors, rather than characteristics specific to aPD (e.g., age, magnitude of levodopa responsiveness), may aid in identifying the most appropriate candidates for DAT [38]. A Delphi analysis conducted by a panel of movement disorder specialists proposed the "5:2:1 rule" as an objective framework for determining when patients inadequately controlled with oral therapy should transition to DAT. According to this guideline, patients meeting the following criteria should be considered for DAT: (1) requiring 5 daily doses or more of oral levodopa; (2) experiencing at least 2 h of "off" symptoms per waking day and/or (3) enduring at least 1 h per day of troublesome dyskinesia [71].

## Patient selection and initiation of CSAI

Within the DAT alternatives, continuous apomorphine infusion has shown to be well tolerated and patients can benefit from it for many years, being the only dopamine analog with overlapping effectiveness to levodopa [38]. Despite the proven efficacy and safety, apomorphine infusion therapy is underused, probably due to difficulties in treatment initiation. Ideal candidates are those with predictable, frequent off periods who retain responsiveness to levodopa but cannot maintain consistent symptom control with oral medications alone. Pre-treatment assessment should include evaluation of cognitive status, psychiatric history, and the presence of impulse control disorders, as these may be exacerbated by dopaminergic stimulation [72]. Additionally, apomorphine’s emetogenic potential may require concurrent antiemetic therapy during initiation. A test dose under medical supervision may help to assess efficacy and tolerability, ensuring safe and effective long-term use.

**Fig. 2:**
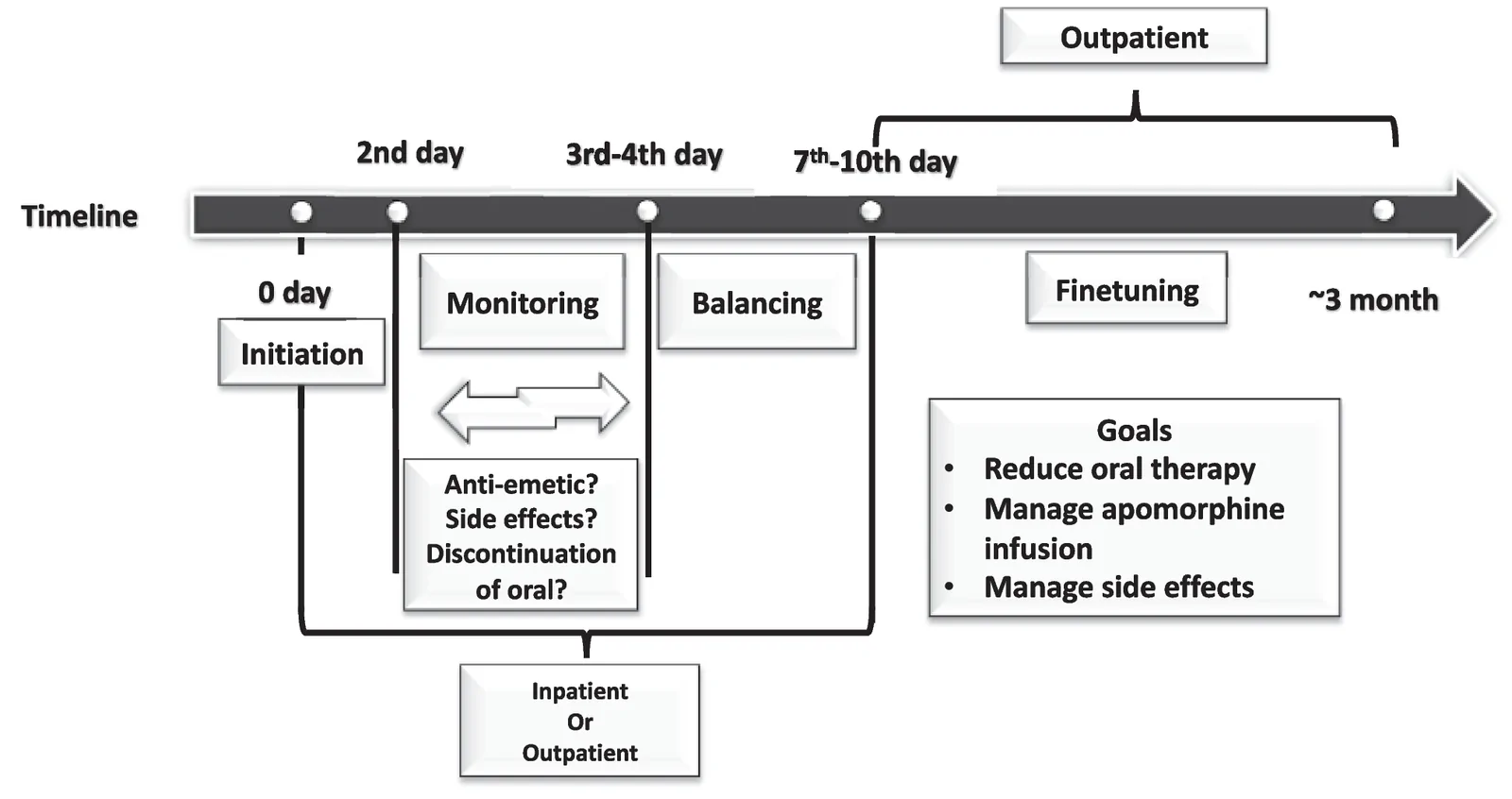
The approach for CSAI initiation includes a stepwise protocol, taking into account the following steps: i) initiation setting; ii) antiemetic treatment, iii) establishment of apomorphine stable dose; iv) amelioration of concomitant anti PD medications; v) bolus dose function; vi) monitoring of patients and management of adverse events

In this complex scenario, we propose an approach that takes into account the evidence present in the literature as well as our opinion as a panel of experts. The approach (Fig. 2) for therapy initiation includes a protocol according to the following steps: i) initiation setting; ii) evaluating the need for antiemetic treatment, iii) establishment of apomorphine stable dose; iv) amelioration of concomitant anti PD medications; v) bolus dose function; vi) monitoring of patients and management of adverse events [72]. Moreover, a recent consensus paper defined the eligibility for device-aided therapies based on (i) motor symptoms—moderate troublesome motor fluctuations, more than 1 h of troublesome dyskinesia per day, more than 2 h of "off" symptoms per day, and more than 5 oral levodopa doses per day; (ii) non-motor symptoms—mild dementia and persistent troublesome hallucinations; (iii) functional impairment—repeated falls despite optimal treatment and difficulty with activities of daily living [71].

The initiation setting should take into consideration the patients characteristics and in some cases, hospital admission may be required. In fact, apomorphine infusion is straightforward to administer but to be successful it requires concordance from the patient and family, and clinical support from an experienced team of doctors and nurses, particularly in the early months of treatment. Patient’s characteristics to be considered are age (> 75 years old is an exclusion criterion for some advanced therapies), comorbidities, and body mass index (BMI, since a low BMI may contraindicate subcutaneous therapies. Furthermore, social and cultural characteristics may influence the decision process, as the infusion device may impact the patient’s quality of life. Luckily, the new devices are smaller and more user friendly and may also be used during work and daily activities. Moreover, neuropsychiatric adverse events represent a clinically relevant concern during CSAI initiation: hallucinations have been reported in approximately 20% of patients at one month and in up to 26% at six months following CSAI initiation. However, this should not be considered an absolute barrier to therapy initiation, as in most cases hallucinations can be effectively managed through dose reduction or the addition of low-dose clozapine, allowing the majority of patients to continue treatment successfully [67]. Ultimately, the caregiver supports the patient receive may be of relevance, especially during the initiation phase. Overall, the initiation phase is usually completed over 5–10 days, whereas further adaptations may require a few more weeks.

The first use of apomorphine was a subcutaneous shot with a penject and was commonly associated with nausea and vomiting due to the dopaminergic activity and may necessitate the use of prophylactic antiemetic therapy. This approach was then translated when the use of slow subcutaneous continuous infusion was started. Domperidone is the most commonly antiemetic drug used for this purpose, as it does not cross the blood–brain barrier and therefore minimizes the risk of central dopaminergic side effects. Typically, domperidone is started at a dose of 10–20 mg three times daily, beginning 2–3 days prior to apomorphine initiation and continued for the first few weeks of treatment [72]. Other centrally acting antiemetics, such as metoclopramide or prochlorperazine, are generally avoided due to their potential to worsen Parkinsonian symptoms [73]. Careful monitoring is advised during this period, especially in elderly patients or those with cardiac risk factors, due to the potential for QT interval prolongation with domperidone [73]. The goal of antiemetic therapy during apomorphine initiation is to enhance tolerability and ensure successful long-term use of apomorphine infusion. Notably, a recent review supported by clinical experience at many centers suggested that subcutaneous apomorphine treatment can be initiated without antiemetic pretreatment [74]. Moreover, a recent study comparing apomorphine sublingual film to subcutaneous apomorphine for OFF episodes in PD demonstrated how the incidence of nausea is not influenced by the preventive use of anti-emetic [75]. In our opinion anti emetic domperidone treatment should be reserved to patients who may be considered at higher risk due to gastroparesis or in case of previous nausea and vomiting reports arising from antiparkinsonian medications for motor symptoms [72, 76]. In this case, anti-emetic domperidone treatment, may be started at least 2 days prior to initiating apomorphine, at a dose of 10 mg three times/day and continued for the shortest possible time [77].

Achieving a stable therapeutic dose is paramount following the initiation phase, necessitating a flexible titration approach balanced between efficacy and patient tolerability. Common strategies include rapid titration, typically inpatient, commencing at 0.5–1.0 mg/hour with increments of 0.5–1.0 mg every few hours or daily, guided by tolerability. Alternatively, a slower, outpatient titration involves 1.0 mg/hour increments over several days. The careful use of anti-emetic treatment is contingent on individual patient response and can significantly influence the intervals required to establish a stable dose by mitigating adverse effects and improving compliance [72].

In our clinical experience, when apomorphine infusion is started dopamine agonists are withdrawn and levodopa therapy is progressively reduced to achieve a more constant dopaminergic stimulation. MAO and COMT inhibitors may be continued along with levodopa if it is still used. It is overall recognized that, once stable infusion is achieved, an optimization of concomitant anti PD medications should be achieved. The Levodopa dose may be reduced by extending dose interval and reducing the dose of each intake. Reducing levodopa favors the improvement of dyskinesia. The final goal is to reduce fluctuations, simplifying the treatment regimen.

For advanced therapy to be successful the role of care-givers is fundamental. In fact the support for technical issues but also a positive psychological support to the patient is crucial to avoid early drop-outs from these therapies.

CSAI is usually well tolerated, and side effects are mostly related to the route of administration. Common adverse effects include nausea, orthostatic hypotension, injection site reactions, and somnolence, particularly during the initial titration period. Long-term use may lead to the development of subcutaneous nodules, which can be minimized by rotating infusion sites and using appropriate needle sizes and skin care techniques. In fact, skin nodules and irritation are rarely a cause for discontinuation and some precautions can be taken to avoid their insurgence, such as massaging the area, using thinner needles, careful cleansing of the area or, in most severe cases, hydrocortisone injections [78]. Cardiovascular monitoring is advised, as apomorphine may exacerbate hypotension, particularly when used in conjunction with antihypertensive medications. Regular follow-up and individualized dose adjustments are critical to balancing efficacy and tolerability. Moreover, although rare, there have been reports of hemolytic anemia associated with long-term apomorphine infusion therapy, making periodic hematological monitoring advisable [64]. In such cases, a direct antiglobulin (Coombs) test may be utilized to evaluate for immune-mediated hemolysis [79]. While the exact mechanism by which apomorphine might trigger this response is not fully understood, it is hypothesized to involve immune modulation in susceptible individuals. Clinicians should be alert to signs of anemia—such as fatigue, pallor, or dyspnea—and investigate appropriately. If hemolytic anemia is confirmed and linked to apomorphine therapy, discontinuation of the drug may be necessary. Therefore, regular complete blood counts and Coombs testing should be considered as part of the long-term safety monitoring protocol in patients receiving CSAI. Overall, with vigilant clinical supervision and patient education, apomorphine infusion remains a safe and effective therapy for managing motor fluctuations in aPD. In our experience communication greatly improves the outcome, with the treating neurologist playing a key role in patient follow-up.

## Expert opinion

Among device-assisted therapies, continuous apomorphine infusion demonstrates excellent tolerability and can provide long-term benefits for patients, standing as the sole dopamine analog with efficacy comparable to levodopa [35]. This unique characteristic positions apomorphine as a particularly valuable therapeutic option, offering robust anti-parkinsonian effects while maintaining a favorable safety profile that allows for sustained use over many years.

The advantages of continuous apomorphine infusion are multifaceted and clinically significant. First and foremost, the continuous dopaminergic stimulation provided by the infusion pump enables remarkable stabilization of motor symptoms, effectively reducing the burden of unpredictable off periods that severely impact patients' quality of life. Unlike oral medications with pulsatile pharmacokinetics, apomorphine infusion delivers steady drug levels throughout the day, mimicking more physiological dopaminergic stimulation. This translates into improved motor function, enhanced independence in activities of daily living, and greater confidence for patients in planning their daily activities.

Furthermore, apomorphine infusion offers significant advantages in managing troublesome dyskinesias. By establishing more stable dopaminergic tone and allowing for strategic levodopa reduction, many patients experience substantial improvement in both peak-dose and biphasic dyskinesias. This dual benefit—better "on" time quality combined with reduced involuntary movements—represents a therapeutic achievement often difficult to obtain with oral medication adjustments alone.

Another compelling advantage lies in treatment simplification. aPD often necessitates complex polypharmacy regimens with multiple daily doses, creating pill burden and increasing the risk of medication errors and non-adherence. Apomorphine infusion consolidates therapy into a single continuous delivery system, dramatically simplifying the treatment regimen while simultaneously improving symptom control. This simplification extends benefits not only to patients but also to caregivers, reducing the stress associated with managing multiple medication schedules.

The flexibility of apomorphine infusion deserves particular emphasis. Doses can be readily adjusted to match individual patient needs and can be titrated relatively quickly compared to some other device-assisted therapies. The subcutaneous route of administration, while requiring initial training, is less invasive than surgical interventions and can be managed in the home setting, preserving patient autonomy and reducing healthcare costs associated with frequent clinic visits. Additionally, the reversibility of the therapy—it can be discontinued or modified without permanent consequences—provides both patients and clinicians with therapeutic confidence and adaptability.

The drug's safety profile relies on appropriate patient selection, proper dose titration, and proactive management of potential complications. Optimal candidates include patients experiencing predictable, frequent off periods who respond well to levodopa but struggle to achieve stable symptom control with oral medications. Prior to treatment, clinicians should assess cognitive function and psychiatric background. While impulse control disorders warrant monitoring, they do not automatically disqualify patients—some may actually improve with continuous dopaminergic stimulation. Additional considerations include age (some centers exclude patients over 75), comorbidities, and body mass index (low BMI may preclude subcutaneous treatments). Caregiver availability is particularly important during treatment initiation, which typically spans 5–10 days, with further adjustments potentially requiring several additional weeks.

Apomorphine can induce nausea and sometimes vomiting especially when administered as an acute injection, the situation change when the drug is infused slowly with a pump starting with low dose. However some physician prefer a premedication with domperidone (the drug is not available in USA). Domperidone is the most commonly antiemetic drug used for this purpose, as it does not cross the blood–brain barrier. It is a potent peripheral antidopaminergic drug therefore minimize dopaminergic side effects such as nausea and vomiting. As reported above, a recent review supported by clinical experience at many centers suggested that subcutaneous apomorphine treatment can be initiated without antiemetic pretreatment [74]. Moreover, a recent study comparing apomorphine sublingual film to subcutaneous apomorphine for OFF episodes in PD demonstrated how the incidence of nausea is not influenced by the preventive use of anti-emetic [75]. In our opinion anti emetic domperidone treatment should be reserved to patients who may be considered at higher risk due to gastroparesis or in case of previous nausea and vomiting reports arising from antiparkinsonian medications for motor symptoms [72, 76]. In this case, anti-emetic domperidone treatment, may be started at least 2 days prior to initiating apomorphine, at a dose of 10 mg three times/day and continued for the shortest possible time [77].

The dose titration can be conducted in out patients clinic or in hospital. It can rapidly achives the best dose which range between 4 and 8 mg/hr.

In our practice, initiating apomorphine infusion involves discontinuing dopamine agonists and gradually reducing levodopa to establish more consistent dopaminergic stimulation. Levodopa reduction occurs through extended dosing intervals and decreased individual doses, which helps improve dyskinesia. The ultimate objective is minimizing fluctuations while simplifying the treatment regimen. In our clinical experience, patients often report not only improved motor control but also enhanced quality of life, greater social engagement, and restored confidence in managing their condition. The psychological impact of regaining predictability in daily functioning cannot be overstated—many patients describe feeling they have "reclaimed their lives" after initiating apomorphine therapy.

Our experience confirms that effective communication significantly enhances outcomes, with the treating neurologist serving a vital role in ongoing patient care. Establishing realistic expectations, providing comprehensive education about the therapy, and maintaining accessible support throughout treatment are essential components that maximize the considerable advantages apomorphine infusion offers to appropriately selected patients with aPD.

## Conclusion

One of the major challenges in the management of aPD is the definition of appropriate timing for introducing advanced therapies, either DAT or DBS. They should be introduced before severe fluctuations or loss of functional independence is reached, when disability can still be improved by dopaminergic treatment optimization.

It is acknowledged that important gaps exist in the different effects DAT and DBS may exert on the patients, especially when considering non-motor aspects of PD or long-term complications. The approach should be realistic, without increasing patients’ and caregivers’ expectations [28, 44].

Generally, patients presenting with levodopa-unresponsive symptoms are not appropriate for DAT selection. Severe neuropsychiatric disturbances, autonomic dysfunction and severe cognitive deficits usually are relative contraindications. One should always assess for any physical, social, or cognitive barriers that could affect the ability to go through the procedure, follow-up, or manage the device appropriately [28, 44].

Future perspectives in aPD management will include the new DAT development, novel DBS systems as well as stem cell transplantation and gene therapy [80]. Most of the innovation in current DAT involves improvements in drug delivery as well as pump systems. In the meantime, CSAI continues to document good efficacy and sustained benefit over time Current initiations protocols take into consideration adverse effects minimization and allow flexibility to reach an optimal titration strategy. This approach can allow neurologists to better manage aPD, thus avoiding sudden interruptions in treatment and improve patients’ quality of life.

Future research is essential to enhance treatment response and further validate the efficacy of various therapeutic options. Such research is crucial for addressing current knowledge gaps regarding the distinctions between available treatments, thereby refining clinical decision-making and guiding clinicians on managing potential treatment discontinuation.
