# Correction to: Device aided therapies in Parkinson disease: an expert view on apomorphine

**DOI:** 10.1007/s10072-026-09407-4

**Published:** 2026-09-18

**Authors:** Fabrizio Stocchi, Roberto Ceravolo, Carlo Colosimo, Maria Francesca De Pandis, Nicola Modugno, Manuela Pilleri, Alessandro Tessitore, Michele Tinazzi, Franco Valzania, Carmine Vitale, Roberta Zangaglia, Mario Zappia, Maurizio Zibetti, Angelo Antonini

**Affiliations:** 1 Department of Neurology, University San Raffaele, Rome, Italy; 2 Institute for Research and Medical Care, IRCCS San Raffaele, Rome, Italy; 3 Department of Clinical and Experimental Medicine, Neurology Unit, University of Pisa, Pisa, Italy; 4 Department of Neurology, Santa Maria University Hospital, Terni, Italy; 5 IRCCS San Raffaele Roma, Cassino, Italy; 6 Department of Human Science and Promotion of Quality of Life, San Raffaele University, Rome, Italy; 7 I.R.C.C.S. Neuromed, Pozzilli, Isernia Italy; 8 UO Neurologia Casa Di Cura Villa Margherita, Arcugnano, Vicenza, Italy; 9 Centro Parkinson E Parkinsonismi, ASST Gaetano Pini CTO, Milan, Italy; 10 Department of Advanced Medical and Surgical Sciences, University of Campania “L. Vanvitelli”, Naples, Italy; 11 Department of Neurosciences, Biomedicine and Movement Sciences, University of Verona, Verona, Italy; 12 Neuromotor and Rehabilitation Department, Neurology Unit, Azienda AUSL-IRCCS Di Reggio Emilia, Reggio Emilia, Italy; 13 Department of Medical, Motor and Wellness Sciences, University of Naples “Parthenope”, Naples, Italy; 14 Parkinson’s and Movement Disorders Center, ICS Maugeri Hermitage, Naples, Italy; 15 Parkinson’s Disease and Movement Disorders Unit, National Institute of Neurology, IRCCS “C. Mondino Foundation”, Pavia, Italy; 16 Department of Medical, Surgical Sciences and Advanced Technologies “GF Ingrassia”, University of Catania, Catania, Italy; 17 Department of Neuroscience “Rita Levi Montalcini”, University of Turin, Via Cherasco 15, 10126 Turin, Italy; 18 SC Neurologia 2U, A.O.U, Città Della Salute E Della Scienza Di Torino, Corso Bramante 88, 10126 Turin, Italy; 19 Neurodegenerative Disease Unit, Department of Neuroscience, Padua Neuroscience Center (PNC), University of Padua, Padua, Italy; 20 IRCCS, San Camillo Hospital, Venice, Italy

**Correction to: Neurological Sciences (2026) 47:630**

**https://doi.org/10.1007/s10072-026-09228-5**

In the original version of the paper, one of the co-authors had passed away before publication, which was unknown to the authors during the final stages of the publication process. As Dr. Manuela Pilleri made a substantial contribution to the work, the authors wish to retain her as a co-author.

The following article note will be added to the front matter:

"In memory of Manuela Pilleri."

In addition, the surname of Dr. Manuela Pilleri should be corrected, as one “l” is missing in the published version.

The original article has been corrected.

